# Supplementary material for: KPNB1 mediates PER/CRY nuclear translocation and circadian clock function
Source: eLife. 2015 Aug 29;4:e08647. doi: 10.7554/eLife.08647 (PMC4597257; doi:10.7554/eLife.08647)
Supplement: Supplementary file 1. — Effects of downregulating ketel genes on free-running circadian locomotor rhythms. DOI: http://dx.doi.org/10.7554/eLife.08647.018 [file elife08647s001.pdf]

Supplementary File 1

Genotype	n	Rhythmic Flies (%) [1]	Period (hr) ± SEM	Power (FFT) ± SEM
ketel RNAi/+ ; dicer/+	16	100	23.22 ± 0.04	0.112 ± 0.011
Pdf-GAL4/+ ; dicer/+	26	100	23.85 ± 0.05	0.059 ± 0.005
Pdf-GAL4/ketel RNAi; dicer/+	21	38.1	23.35 ± 0.15	0.022 ± 0.004[2]
TUG/+ ; dicer/+	26	100	24.03 ± 0.06	0.119 ± 0.009
TUG/ketel RNAi; dicer/+	19	0	-	-
ketel RNAi/+ ; *Pdf*-GS/dicer (+EtOH)	16	100	23.68 ± 0.04	0.039 ± 0.004
ketel RNAi/+ ; *Pdf*-GS/dicer (+RU486)	16	18.7	23.75 ± 0.09	0.014 ± 0.001[3]

1 Flies with FFT value > 0.01 are counted as a rhythmic.

2 P < 0.001 compared to both ketel RNAi/+ ; dicer/+ and *Pdf*-GAL4/+ ; dicer/+ controls, by Student's t-test

3 The flies became arrhythmic 6-7 days after transferring to DD. Periods and FFTs after 7 days in DD are shown. P < 0.05 compared to EtOH-treated control flies, by Student's t-test
